# Effect of Comprehensive Balance Modulating Strategies on Physical Performance Among the Elderly in Care Home Settings in Southern India

**DOI:** 10.7759/cureus.67373

**Published:** 2024-08-21

**Authors:** Binoy Mathew K. V., Jagatheesan Alagesan, Prathap Suganthirababu

**Affiliations:** 1 Physiotherapy, Saveetha College of Physiotherapy, Saveetha Institute of Medical and Technical Sciences, Chennai, IND

**Keywords:** four step square test (fsst), short physical performance battery, balance training in geriatrics, institutionalized elderly, seniors, randomised controlled trial, physical functional performance, exercise training, postural balance, aged adult

## Abstract

Purpose

Globally, the proportion of the elderly population is rising. Age-related physical performance impairments are more common and affect quality of life. This study aimed to investigate the impact of a new exercise regimen called Comprehensive Balance-Modulating Strategies (CBMS) on the physical performance of older adults living in care home settings.

Methods

Forty-eight functionally independent elderly individuals were randomized into two groups: group A received the CBMS programme for eight weeks, and group B received routine medical care. The Short Physical Performance Battery (SPPB) and the Four Square Step Test (FSST) were outcome measures. Outcomes were measured at baseline, immediately after the intervention, and eight weeks after the intervention.

Results

The mean and standard deviation of subjects’ ages in both groups were 72.46 (8.28) and 68.12 (6.95), respectively. The CBMS programme significantly improved physical performance among the intervention group (*p* = <0.0001).

Conclusion

The present study found that CBMS was effective in improving physical performance among institutionalized elderly. Large-scale clinical trials and research exploring the effect of CBMS among community-dwelling elderly individuals are recommended.

## Introduction

The percentage of elderly individuals in most countries is rising, indicating that the global population is ageing quickly. By 2050, this percentage is predicted to reach 22% of the total population [1]. The elderly, who function well physically, are better able to accomplish daily tasks and have higher levels of life satisfaction [2].

The incidence of physical performance restrictions also increases with the age of the population, which has a negative impact on quality of life and increases medical costs. It was found that almost 40% of people 60 years of age and older had trouble getting out of a chair and slowed their gait. Poor physical performance has been linked to deficits in motor coordination, balance, and the functioning of the skeletal, muscular, and neurological systems, in addition to age-related loss of muscle strength [3]. Among older adults living in institutional care settings, these deficits in physical and functional abilities are noticeably more prevalent [4]. In addition, a sedentary lifestyle impairs balance control and hastens the deterioration of physical function. The results of a systematic review supported this finding, showing that most randomized controlled trials comparing different physical training regimens to improve static balance among the elderly showed a deterioration in physical function among the elderly in the control groups [5].

Physical performance evaluation helps identify senior citizens at risk of falling and develop suitable preventive measures. The usual gait speed, Short Physical Performance Battery (SPPB), six-minute walk test, timed-up and go test, and 400-meter walking test are the most commonly used methods for evaluating physical performance [6]. Chair stand time and gait speed were used as outcome measures of physical performance in the Framingham Offspring Study [7]. Walking speed is a highly reliable indicator of general health and the preservation of physical and functional abilities [8]. The relationship between normal gait speed and mood, motivation, musculoskeletal health, cognitive function, and cardiopulmonary fitness shows that gait speed should be considered the sixth vital sign [9]. Increased mortality has been reported in patients with poor performance in evaluation tests measuring walking speed, standing balance, chair rise test, and grip strength [10,11].

To promote healthy ageing, it is crucial to prevent and treat physical performance deterioration in elderly individuals. Impaired motor coordination and balance can be improved through appropriate exercises. Seniors who exercise report improvements in physical and cognitive function based on a systematic review and meta-analysis [12]. In older adults, physical activity improves muscle mass, muscle strength, balance, and functional independence. Another systematic review discovered that among healthy seniors, physical activity improves physical performance further, resulting in significant changes in gait speed, chair rise time, and balance [13]. It has been stated that the best intervention for fall prevention is balance exercise. Since standing upright and active functional activities depend on balance, balance training should be considered [5,14]. The usefulness of balancing training as a fall prevention and physical performance-enhancing method was demonstrated by the fact that elderly individuals with balance deficits experienced more falls than those elderly with greater balance control [15].

Because elderly people tend to be less fit than younger adults, it is recommended that training HRs (heart rates) be maintained between 40% and 50% of maximal HR as a safety precaution to prevent unfavourable outcomes [16]. The type of exercise most effective in improving the physical performance of older adults is still debated [17,18]. Therefore, to assess the impact of a novel balancing regimen known as Comprehensive Balance Modulating Strategies (CBMS) on physical performance among the elderly in institutionalized settings, we conducted a randomized controlled trial.

## Materials and methods

**Figure 1:**
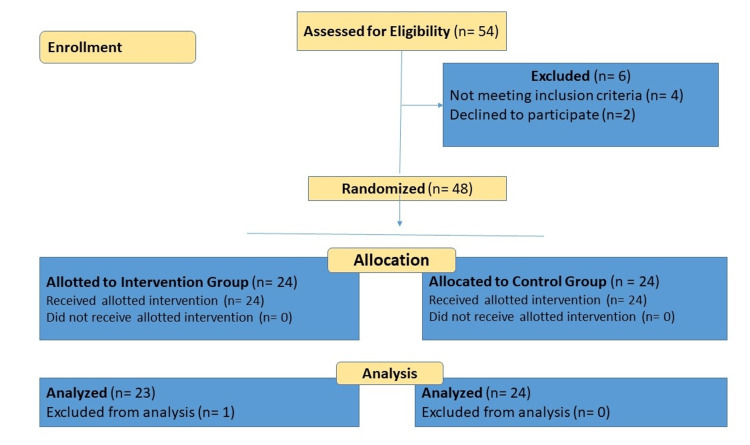
CONSORT flow chart CONSORT: Consolidated Standards of Reporting Trials

This was a randomized clinical trial with a parallel group design that was applied, applying the Consolidated Standards of Reporting Trials guidelines (CONSORT) [19], as depicted in Figure 1. Between December 2022 and March 2023, the study was conducted in compliance with the Helsinki Declaration and COVID-19 principles and safeguards. Before the research project began, Institutional Ethical Committee approval (Ref. No. IEC/2021/04/03) was secured. The trial was registered in the Clinical Trial Registry of India (CTRI) on March 2, 2022, with registration number Trial CTRI/2022/03/040729. The subjects provided written informed consent before the start of the study.

Participants

Participants in the study were older adults, regardless of gender, over sixty, who could walk without assistance. The exclusion criteria were vestibular abnormalities, cognitive impairment, and medical diseases that could prevent regular exercise participation. The sample size was determined from a pilot study involving 20 participants. After performing the sample size calculation using the pilot study data, we determined that a minimum sample size of 21 participants was required for each group in the main study.

In Kerala, the southern Indian state, the study involved 48 senior citizens living in four institutional settings. Random assignments were made to the intervention or control groups for each subject using a computer-generated random number table.

Procedure

The intervention group received a validated balanced exercise regimen called the CBMS (Table 1). The intervention consisted of 20 minutes of supervised exercise provided three times a week for eight weeks. The control group received regular health examinations and medical care.

**Table 1:** Comprehensive Balance Modulating Strategies (CBMS) exercise protocol

Time duration	Exercise components
Warm up: 5 minutes. All the exercises are in upright standing.	(1) Raise arms upwards with inspiration and arms down with expiration (10 repetitions). (2) Hold arms front at shoulder level, widen arms away with inspiration, and bring arms back with expiration (10 repetitions).(3) Trunk rotation with arms crossed at chest (5 repetitions to each side). (4) March with legs up in the same place (can take support from the window or wall if needed) (1 minute).
Balance exercises: 20 minutes	(1) Forward walking: 2 minutes (rest for 1 minute). (2) Backward walking: minimal support and guidance can be provided if needed. 2 minutes (rest for 1 minute). (3) Sidewalking to the right side (minimal support and guidance can be provided if needed) 2 minutes (rest for 1 minute). (4) Sidewalking to the left side (minimal support and guidance can be provided if needed) 2 minutes (rest for 1 minute). (5) Toe walking (those unable to do so can do forward walking instead) 2 minutes (rest for 1 minute). (6) Heel walking on heels (those unable to do so can do backward walking instead) 2 minutes (rest for 1 minute). (7) Single leg stance: on alternate leg (maximum time up to 10 seconds) If not possible, you can do marching in standing with window or wall support for 2 minutes.
Cool down: 5 minutes. All the exercises are in upright standing.	(1) Raise arms upwards with inspiration and arms down with expiration (10 repetitions). (2) Hold arms front at shoulder level, widen arms away with inspiration, and bring arms back with expiration (10 repetitions). (3) Trunk rotation with arms crossed at chest (5 repetitions to each side). (4) March with legs up in the same place (can take support from the window or wall if needed) (1 minute).

Due to the nature of the interventions, there was no blinding of the participants, investigator, or assessor. The lead investigator, with the support of an assistant, conducted the assessments and interventions.

Outcome measures

The Four Square Step Test (FSST) and the SPPB were used as assessment instruments to evaluate physical performance. A certified physiotherapist who was not a part of the research team assisted a researcher in conducting the physical performance assessment at baseline, immediately after the eight-week intervention, and eight weeks later.

SPPB

The three-part SPPB is an evaluation tool. The test is divided into sections to assess sit-to-stand ability, gait speed, and balance. Excellent predictive validity, test-retest reliability, and clinical applicability characterize SPPB. Five chair-stand tests, three or four meters of walking at a regular speed, and an increasing assessment of standing balance are all included in the SPPB. The three-part assessment tool has four scoring categories, ranging from 0 to 4. When all elements are added together, a score between 0 and 12 is obtained. Low performers have scores between 0 and 6, intermediate performers have scores between 7 and 9, and top performers have scores between 10 and 12 [20]. A change of 0.5 points is considered a small meaningful change, whereas a change of 1 point is considered a substantial meaningful change [21].

FSST

Dite and Temple created the FSST to evaluate dynamic balance and quick direction changes. The test was administered twice, with the best time being considered. If the task takes more than 15 seconds to finish, there is a deficit in dynamic balance and a higher chance of falling [22].

Statistical analysis

The baseline properties of the data were evaluated using descriptive statistics. Quantitative variables were presented as means and standard deviations, or medians and interquartile ranges, depending on the normality of the data; counts and percentages were used to describe qualitative factors. Depending on the data's normalcy, independent t-tests or Mann-Whitney U tests were used to compare quantitative parameters between the study groups. Repeated measures analysis of variance or Friedman tests were used to compare groups within the pretest, posttest, and follow-up periods. P-values less than 0.05 with a 95% confidence interval were used to evaluate statistical significance. Version 20.0 of SPSS software (IBM Corp., Armonk, NY) was used to analyze the data processed in Microsoft Excel (Microsoft® Corp., Redmond, WA).

## Results

Participant demographics

The mean and standard deviation of subjects’ ages in both intervention and control groups were 72.46 (8.28) and 68.12 (6.95) years old, respectively. In the intervention group (n = 24), 14 were male, and in the control group (n = 14), 11 were male. 

Table 2 illustrates the comparison of the total balance score subunit of SPPB for both groups at three time intervals. Table 3 reports the comparison of gait speed scores in SPPB. The comparison of repeated chair stand test scores of SPPB is in Table 4, and the total score comparison of SPPB is in Table 5, respectively.

**Table 2:** Total balance score comparison across pre-test, post-test, and follow-up time points within CBMS and control groups Friedman test, p<0.05, shows the significance. SD: standard deviation; IQR: interquartile range; CBMS: Comprehensive Balance-Modulating Strategies. *Significance, p-value < 0.05.

Total balance score	Overall			Intervention group			Control group		
	Range	Mean ±SD	Median (IQR)	Range	Mean ±SD	Median (IQR)	Range	Mean ±SD	Median (IQR)
Pre-test	0–4	2.81 ± 1.25	3(2‒4)	0–4	2.38 ± 1.21	2.5(1‒3)	0–4	3.25 ± 1.15	4(2‒4)
Post-test	0–4	3.29 ± 1.03	4(2.25‒4)	0–4	3.42 ± 0.93	4(3‒4)	0–4	3.17 ± 1.13	4(2‒4)
Follow up	0–4	3.1 ± 1.08	3.5(2‒4)	0–4	3.04 ± 1.04	3(2.25‒4)	0–4	3.17 ± 1.13	4(2‒4)
Test statistic (Sig.)	20.258 (<0.0001)*			28.43 (<0.0001)*			4 (0.135)		

**Table 3:** Gait Speed Score comparison across pre-test, post-test, and follow-up time points within CBMS and control groups Friedman test, p < 0.05, shows the significance. SD: standard deviation; IQR: interquartile range; CBMS: Comprehensive Balance-Modulating Strategies. *Significance, p-value < 0.05.

Gait Speed Score	Overall			Intervention group			Control group		
	Range	Mean ± SD	Median (IQR)	Range	Mean ± SD	Median (IQR)	Range	Mean ± SD	Median (IQR)
Pre-test	0–4	2.67 ± 0.97	3(2‒3)	0–4	2.46 ± 0.83	2.5(2‒3)	0–4	2.88 ± 1.08	3(2‒4)
Post-test	0–4	2.92 ± 0.94	3(2‒4)	0–4	3.17 ± 0.87	3(3‒4)	0–4	2.67 ± 0.96	3(2‒3)
Follow up	0–4	2.69 ± 0.95	3(2‒3)	0–4	2.83 ± 0.87	3(2‒3.75)	0–4	2.54 ± 1.02	2.5(2‒3)
Test statistic (Sig.)	7.792 (0.020)*			21.043 (<0.0001)*			8 (0.018)*		

**Table 4:** Repeated Chair-Stand Test Score comparison across pre-test, post-test, and follow-up time points within CBMS and control groups Friedman test, p < 0.05, shows the significance. SD: standard deviation; IQR: interquartile range; CBMS: Comprehensive Balance-Modulating Strategies. *Significance, p-value < 0.05.

Repeated chair stand test	Overall			Intervention group			Control group		
	Range	Mean ± SD	Median (IQR)	Range	Mean ± SD	Median (IQR)	Range	Mean ± SD	Median (IQR)
Pre-test	0‒4	1.5 ± 1.13	1(1‒2)	0‒4	1.13 ± 0.95	1(0‒2)	0‒4	1.88 ± 1.19	2(1‒3)
Post-test	0‒4	1.63 ± 1.16	2(1‒2)	0‒4	1.58 ± 1.21	1.5(1‒2)	0‒4	1.67 ± 1.13	2(1‒2)
Follow up	0‒4	1.51 ± 1.08	1(1‒2)	0‒4	1.39 ± 1.03	1(1‒2)	0‒4	1.63 ± 1.13	2(1‒2)
Test statistic (Sig.)	2.851(0.024)*			13(0.002)*			8.857(0.012)*		

**Table 5:** SPPB total score comparison across pre-test, post-test, and follow-up time points within CBMS and control groups The Friedman test, p<0.05, shows the significance. SD: standard deviation; IQR: interquartile range; CBMS: Comprehensive Balance-Modulating Strategies. *Significance, p-value < 0.05.

SPPB total score	Overall			Intervention group			Control group		
	Range	Mean ±SD	Median (IQR)	Range	Mean ±SD	Median (IQR)	Range	Mean ±SD	Median (IQR)
Pre-test	0‒12	6.96 ± 2.71	8(5‒9)	0‒12	5.92 ± 2.54	6.5(4‒8)	0‒12	8 ± 2.5	8 (7‒10)
Post-test	0‒12	7.85 ± 2.64	8(6.25‒10)	0‒12	8.21 ± 2.6	8.5(7.25‒10)	0‒12	7.5 ± 2.69	8 (6‒9.75)
Follow up	0‒12	7.26 ± 2.57	7(6‒9)	0‒12	7.17 ± 2.52	7(6‒9)	0‒12	7.33 ± 2.66	7.5 (6‒9.75)
Test statistic (Sig.)	10.903(0.004)*			37.081(<0.0001)*			15.520(<0.0001)*		

 The comparison of FSST comparison is shown in Table 6.

**Table 6:** FSST comparison across pre-test, post-test, and follow-up time points within CBMS and control groups Friedman test, P < 0.05, shows the significance. SD: standard deviation; IQR: interquartile range; CBMS: Comprehensive Balance-Modulating Strategies. *Significance, p-value < 0.05.

FSST faster	Overall			Intervention group			Control group		
	Range	Mean ±SD	Median (IQR)	Range	Mean ±SD	Median (IQR)	Range	Mean ±SD	Median (IQR)
Pre-test	9.06‒33.63	18.26 ±6.33	17.06 (12.75‒23.38)	9.62‒33.63	19.63 ±6.05	20.1 (15.33‒23.4)	9.06‒30.67	16.9 ±6.43	15.12 (11.64‒22.47)
Post-test	9.07‒31.14	17.32 ±5.84	15.43 (12.78‒22.11)	9.07‒28.19	17.05 ±5.16	15.96 (12.91‒21.66)	9.54‒31.14	17.59 ±6.55	14.61 (12.78‒23.41)
Follow up	9.66‒31.63	18.67 ±6.04	17.47 (14.23‒24.23)	9.83‒30.76	18.98 ±5.29	19.45 (14.24‒23.18)	9.66‒31.63	18.37 ±6.77	15.44 (13.2‒24.88)
Test statistic (Sig.)	28.952(<0.0001)*			26.435(<0.0001)*			13.537(0.001)*		

## Discussion

The study findings showed that exercise programs significantly improved the physical performance metrics of most institutionalized elderly people, as determined by the FSST and the SPPB. The current study showed that the total SPPB score for both the intervention and control groups significantly improved, with the intervention group showing more notable improvements. This highlights the significance of customized procedures for maximizing functional outcomes in older people.

The analysis of the SPPB subcomponents provided more detailed information. The efficacy of CBMS was demonstrated by the study, which found significant gains over time in the overall balance component of the SPPB for both the intervention group (p<0.0001) and the cohort as a whole (p<0.0001). However, no significant change was observed in the control group (p=0.135), indicating that there was no discernible improvement in balance over time in the control group. To fully comprehend the precise causes of these improvements and their long-term effects, more research is necessary.

According to the study, improvements in gait speed scores were reported by both the intervention and control groups; however, the intervention group's improvements were only slightly greater than those of the control group. The study revealed that there were significant changes in the repeated chair stand test component of SPPB in both the intervention and control groups, negating any special effect of the CBMS program.

After an eight-week intervention, older adults in the intervention group demonstrated statistically significant improvements in their performance on the FSST compared to the control group. During the follow-up evaluation, the intervention group's average FSST completion time was lower than that of the baseline. In comparison to the intervention group, the control group’s FSST performance also improved, although not significantly.

Compared with the control group, which received conventional normal medical treatment, the overall results demonstrated that the CBMS program effectively brought about significant changes in the elderly living in institutions. Walking forward, walking backward, walking sideways, walking on heels, walking on toes, and single-leg balancing were all included in the intervention program. Humans employ a variety of foot positioning techniques to maintain their balance and avoid falling [23]. The American College of Sports Medicine's recommendations for older individuals, which call for 20 to 30 minutes of exercise two to three days a week to enhance balance, agility, coordination, and gait, served as the foundation for the intervention program [24].

Walking has been shown to lower the risk of falling by maintaining and enhancing muscle strength, balance, coordination, and proprioception. It has been noted that regular outdoor walking helps older people's balance and self-perceived walking speed [25,26]. It is believed that walking and other physical exercise are good ways to support healthy ageing.

Training for backward walking has been shown to enhance the pattern of hip extension during knee flexion. Because both motions involve similar brain regions, training for backward walking can be transferred to forward walking with ease [27]. Research has shown that walking backward increases activity in the thalamus, putamen, caudate nucleus, superior parietal lobule, supplementary motor region, and precentral gyrus. This study demonstrates how reverse walking poses a stability challenge [28,29]. An electroencephalogram study demonstrated that walking backward activates the sensorimotor cortex more than walking forwards [30]. Additionally, backward walking is used to assess different kinds of populations' fall risk, mobility, and balance [31].

It is noted that elderly people have poor lateral balance. Older adults prioritize lateral balance even despite rising energy expenses. Elderly people living in the community showed a considerable increase in gait speed after just six weeks of the sideways walking intervention, which continued for an additional six weeks. Additionally, following the intervention, there was a little drop in step width variability [32]. The intervention group's gait speed and dynamic balance may have improved because of all these factors.

Limitations and recommendations

One of the shortcomings of the current study was the small sample size. Furthermore, because the study was conducted on older people living in institutions, the results cannot be generalized to all older people.

Therefore, comparable large-sample studies and comparable research, including senior citizens who live in the community, are recommended. To determine which exercise program is best for improving physical performance, the one employed in this study should be compared with others.

## Conclusions

Our study aimed to investigate the effectiveness of a new exercise regimen called the CBMS on the physical performance of older adults living in care home settings. The findings revealed that CBMS was effective in enhancing the physical performance of institutionalized elderly people. Significant changes in balance and gait speed were noted in the intervention group.

We recommend conducting extensive clinical trials to examine the impact of CBMS on community-dwelling older adults. Further research is warranted to explore the efficacy of CBMS with various exercise programmes.
